# Geriatric Nutritional Risk Index and Controlling Nutritional Status Score can predict postoperative 180-day mortality in hip fracture surgeries

**DOI:** 10.1186/s40981-019-0282-6

**Published:** 2019-09-25

**Authors:** Atsushi Kotera

**Affiliations:** Department of Anesthesiology, Kumamoto Central Hospital, 955 Muro, Ozu-machi Kikuchi-gun, Kumamoto, 869-1235 Japan

**Keywords:** Hip surgery, Geriatric Nutritional Risk Index, Controlling Nutritional Status Score, Postoperative mortality

## Abstract

**Background:**

The Geriatric Nutritional Risk Index (GNRI) based on serum albumin level and body weight and the Controlling Nutritional Status Score (CONUT) based on serum albumin level, total cholesterol level, and total lymphocyte count were created to evaluate objectively a patient’s nutritional status in 2005. Here we validated the usefulness of the GNRI and the CONUT as a prognostic factor of the 180-day mortality in patients who underwent hip fracture surgeries.

We retrospectively collected data from patients with hip surgeries performed from January 2012 to December 2018. The variables required for the GNRI and the CONUT and the factors presumably associated with postoperative mortality including the patients’ characteristics were collected from the medical charts. Intergroup differences were assessed with the *χ*^2^ test with Yates’ correlation for continuity in category variables. The Mann-Whitney *U* test was used to test for differences in continuous variables. We validated the power of the GNRI and the CONUT values to distinguish patients who died ≤ 180 days post-surgery from those who did not, by calculating the area under the receiver operating characteristic curve (AUC). The correlation between these two models was analyzed by Spearman’s rank correlation (*ρ*).

**Results:**

We retrospectively examined the cases of 607 patients aged 87 ± 6 (range 70–102) years old. The 180-day mortality rate was 5.4% (*n* = 33 non-survivors). The GNRI value in the non-survivors was 83 ± 9 (range 66–111), which was significantly lower than that in the survivors at 92 ± 9 (range 64–120). The CONUT value in the non-survivors was 6 ± 3 (range 1–11), which was significantly higher than that in the survivors at 4 ± 2 (range 0–11). The AUC value to predict the 180-day mortality was 0.74 for the GNRI and 0.72 for the CONUT. The *ρ* value between these two models was 0.61 in the total of 607 patients and was 0.78 in the 33 non-survivors.

**Conclusions:**

Our results suggest that the GNRI and the CONUT are a simple and useful tool to predict the 180-day mortality in patients who have undergone a hip surgery.

## Background

The number of patients with hip fractures is increasing worldwide due to the aging of many populations [1]. Hip fractures can introduce various serious physical complications following prolonged immobility. Thus, the reported mortality rates of patients with hip fractures at 1 month and 6 months are 2.7% and 10.8%, respectively [2]. Several risk factors related to postoperative mortality after hip surgeries have been reported in the previous reports, and malnutrition is noted to be an important risk factor among them [3, 4]. Malnutrition is considered to be associated with a longer hospital stay as well as increased morbidity and mortality [5]. However, the ideal method to evaluate nutritional status in elderly patients with hip fractures is still controversial.

The Geriatric Nutritional Risk Index (GNRI) based on serum albumin level and body weight and the Controlling Nutritional Status Score (CONUT) based on serum albumin level, total cholesterol level, and total lymphocyte count were created to assess a patient’s nutritional status objectively in 2005 [6, 7]. These two models are simple and easy-to-use tools for clinicians. In the present study, we aimed to validate the usefulness of the GNRI and the CONUT as a prognostic factor of the 180-day mortality in patients who underwent hip fracture surgeries over a 7-year period at our hospital.

## Patients and methods

### Patients

The approval for this retrospective study (no. 2017-013) was provided by the Ethical Committee of Kumamoto Central Hospital, Kumamoto, Japan, on December 27, 2017. Patients who underwent osteosynthesis or hip hemiarthroplasty for a hip fracture under general or spinal anesthesia performed between January 1, 2012, and December 31, 2018, at Kumamoto Central Hospital were eligible for this study. All data were obtained from the patients’ medical records without personal information. Informed consent from the patients was therefore waived, based on the Ethical Guidelines for Epidemiological Studies issued jointly by the Ministry of Health, Labour and Welfare and the Ministry of Education, Culture, Sports, Science, and Technology of Japan.

### Data collection

Data were collected based on the variables required for the GNRI and the CONUT. The GNRI requires serum albumin level and body weight, and the CONUT requires serum albumin level, total cholesterol level, and total lymphocyte count. A patient’s body weight was measured on the bed with weight meter automatically at his or her admission day. The GNRI was calculated as follows [6]: GNRI = 14.89 × serum albumin level (g/dl) + 41.7 × present body weight/ideal body weight. The ideal body weight was defined as (height (m))^2^ × 22. GNRI was classified as severe (< 82), moderate (≥ 82, < 92), low (≥ 92, < 99), and normal (≥ 99) [6]. The CONUT was a sum of the scores based on serum albumin (0, 2, 4, 6), total cholesterol level, and total lymphocyte count (0, 1, 2, 3, for each) and classified as severe (≥ 9), moderate (5–8), low (2–4), and normal (0–1) [7].

Factors presumably associated with postoperative complications including patient characteristics were also extracted from the medical charts: patient age, gender, underlying co-morbidities, the ejection fraction measured by echocardiography, the presence or absence of dementia, the waiting period prior to surgery, the method of anesthesia, the precise type of surgery, the operation time, and the anesthesia time. At our hospital, the method of anesthesia is left to the individual anesthesiologist. When the patient’s preoperative condition was stable, general anesthesia was the first choice. Conversely, when the patient had one or more serious underlying co-morbidities such as chronic heart failure or pulmonary disease, spinal anesthesia was administered.

In this study, we set the 180-day mortality as the endpoint, and we collected patients’ biochemical data which was sampled at their hospital admission day. When a patient dies after leaving our hospital, the family doctor, the nursing home staff, or the family always informs us the information. And that is certainly recorded in the patient’s medical chart by our hospital staff. We divided the patients into two groups: those who were still alive at 180 days post-surgery (the survivor group) and those who had not survived as of 180 days post-surgery (the non-survivor group), and we analyzed the patients’ characteristics, the GNRI value, and the CONUT value between the two groups. We also divided the patients into the four groups (severe, moderate, low, and normal) according to the grade of malnutrition using a previously established threshold [6, 7], and we compared the 180-day mortality among the four groups. We assessed the power of the GNRI and CONUT values to distinguish patients who died ≤ 180 days post-surgery from those who did not, by calculating the area under the receiver operating characteristic curve (AUC).

### Statistical analysis

All statistical analyses were carried out using the software program Excel Tokei 2012 (Social Survey Research Information, Tokyo). Intergroup differences were assessed with the *χ*^2^ test with Yates’ correlation for continuity in category variables. The Mann-Whitney *U* test was used to test for differences in continuous variables. Differences of *p* < 0.05 were considered significant. Descriptive data are presented as the mean ± standard deviation (range).

We assessed the power of a model to distinguish patients who died within 180 days after the hip fracture surgery from those who did not by calculating the area under the receiver operating characteristic curve (AUC) [8]. The AUC value ranged from 0.5 to 1.0 and the greater the AUC, the better the model. An AUC of 1.0 indicates a perfect model that has 100% sensitivity and 100% specificity. An AUC of 0.5 indicates a model that is completely ineffective in differentiating between real cases and non-cases. In addition, we analyzed the statistical correlation between these two models by Spearman’s rank correlation (*ρ*).

## Results

During the study period, 635 hip fracture surgeries were undergone. However, 28 patients were excluded because of the lack of blood examinations required for calculating the GNRI or the CONUT. We analyzed the data of 607 patients (Table 1). The 180-day mortality rate was 5.4%. The percentage of male patients in the non-survivors was significantly higher than in the survivors (33.3% vs. 17.9%, *p* = 0.028). The presence of dementia in the non-survivors was significantly higher than in the survivors (66.6% vs. 47.8%, *p* = 0.042). There were no significant differences in other clinical characteristics between these two groups. Serum albumin, ratio of present to ideal body weight, total cholesterol level, and total lymphocyte count were significantly higher in the survivors than in the non-survivors (Table 2). In the non-survivors, the GNRI was significantly lower and the CONUT was significantly higher than in the survivors (*p* = < 0.001 for both).

**Table 1:** Comparison of clinical characteristics between the non-survivors and survivors

	Non-survivors (*n* = 33)	Survivors (*n* = 574)	*p* value
Age (year)	88 ± 7 (73–99)	87 ± 6 (70–102)	0.129
Male	11 (33.3)	103 (17.9)	0.028
Dementia	22 (66.6)	278 (47.8)	0.042
Co-morbidities			
Hypertension	16 (48.5)	306 (53.3)	0.589
Chronic heart failure	7 (24.2)	64 (11.1)	0.083
Old cerebral infarction	6 (18.2)	75 (13.1)	0.401
Diabetes mellitus	3 (9.1)	78 (13.6)	0.460
Chronic renal failure	3 (9.1)	21 (3.7)	0.119
Respiratory related disease*	3 (9.1)	67 (11.7)	0.652
Rheumatoid arthritis	2 (6.1)	10 (1.7)	0.083
Insertion of cardiac pacemaker	2 (6.1)	10 (1.7)	0.083
Old myocardial infarction	1 (3.0)	12 (2.1)	0.717
Ejection fraction (%)	63 ± 12 (25–89)	64 ± 9 (33–88)	0.606
Waiting period for surgery (day)	9 ± 4 (4–18)	9 ± 3 (2–25)	0.931
Type of anesthesia			
General anesthesia	24 (72.7)	407 (70.9)	0.823
Spinal anesthesia	9 (27.3)	167 (29.1)	
Type of surgery			
Hip hemiarthroplasty	8 (29.4)	105 (19.6)	0.393
Osteosynthesis	25 (70.6)	469 (80.4)	
Operation time (min)	47 ± 24 (18–115)	46 ± 24 (13–190)	0.675
Anesthesia time (min)	91 ± 34 (30–173)	88 ± 29 (33–262)	0.634
Intraoperative blood loss (g)	44 ± 61 (10–195)	43 ± 89 (10–860)	0.623

The data are given as patient’s number (%) or the mean ± standard deviation (range). *Respiratory related disease includes chronic obstructive pulmonary disease, bronchial asthma, and pulmonary tuberculosis

**Table 2:** Comparison of the GNRI and CONUT between non-survivors and survivors

	Non-survivors (*n* = 33)	Survivors (*n* = 574)	*p* value
GNRI	83 ± 9 (66–111)	92 ± 9 (64–120)	< 0.001
Serum albumin level (g/dl)	3.2 ± 0.5 (2.2–4.0)	3.5 ± 0.4 (2.1–4.6)	< 0.001
Ratio of the present to the ideal body weight	0.88 ± 0.16 (0.65–1.40)	0.93 ± 0.14 (0.62–1.46)	0.010
CONUT	6 ± 3 (1–11)	4 ± 2 (0–11)	< 0.001
Serum albumin level (mg/dl)	3.2 ± 0.5 (2.2–4.0)	3.5 ± 0.4 (2.1–4.6)	< 0.001
Total cholesterol level (mg/dl)	150 ± 33 (99–225)	170 ± 35 (79–292)	0.002
Total lymphocyte count (/mm^3^)	834 ± 369 (172–2057)	1013 ± 451 (191–3334)	0.023

The data are given as the mean ± standard deviation (range)

*GNRI* Geriatric Nutritional Risk Index, *CONUT* Controlling Nutritional Status Score

The 30-day postoperative complications are listed in Table 3. The rate of postoperative major complications was 29.0% and pneumonia was the most common postoperative complication. The causes of the 180-day mortality were as follows: 18 patients died of pneumonia, 10 patients died of postoperative heart failure, and five patients died of sepsis secondary to urinary tract infection or bile tract infection. The survival days after the surgery in the non-survivors were 86 ± 58 (range 5–178) days.

**Table 3:** Postoperative major complications

Postoperative major complications	*N* (% of the 30-day postoperative complications)
Pneumonia	47 (7.7)
Urinary tract infection	45 (7.4)
Venous thrombus	44 (7.2)
Postoperative heart failure	16 (2.6)
Surgical site infection	12 (2.0
Sepsis	5 (0.8)
Bile tract infection	2 (0.3)
Pseudomembranous enteritis	2 (0.3)
Stroke	2 (0.3)
Acute myocardial infarction	2 (0.3)
Total	176 (29.0)

The data are given as patient’s number (%)

**Fig. 1:**
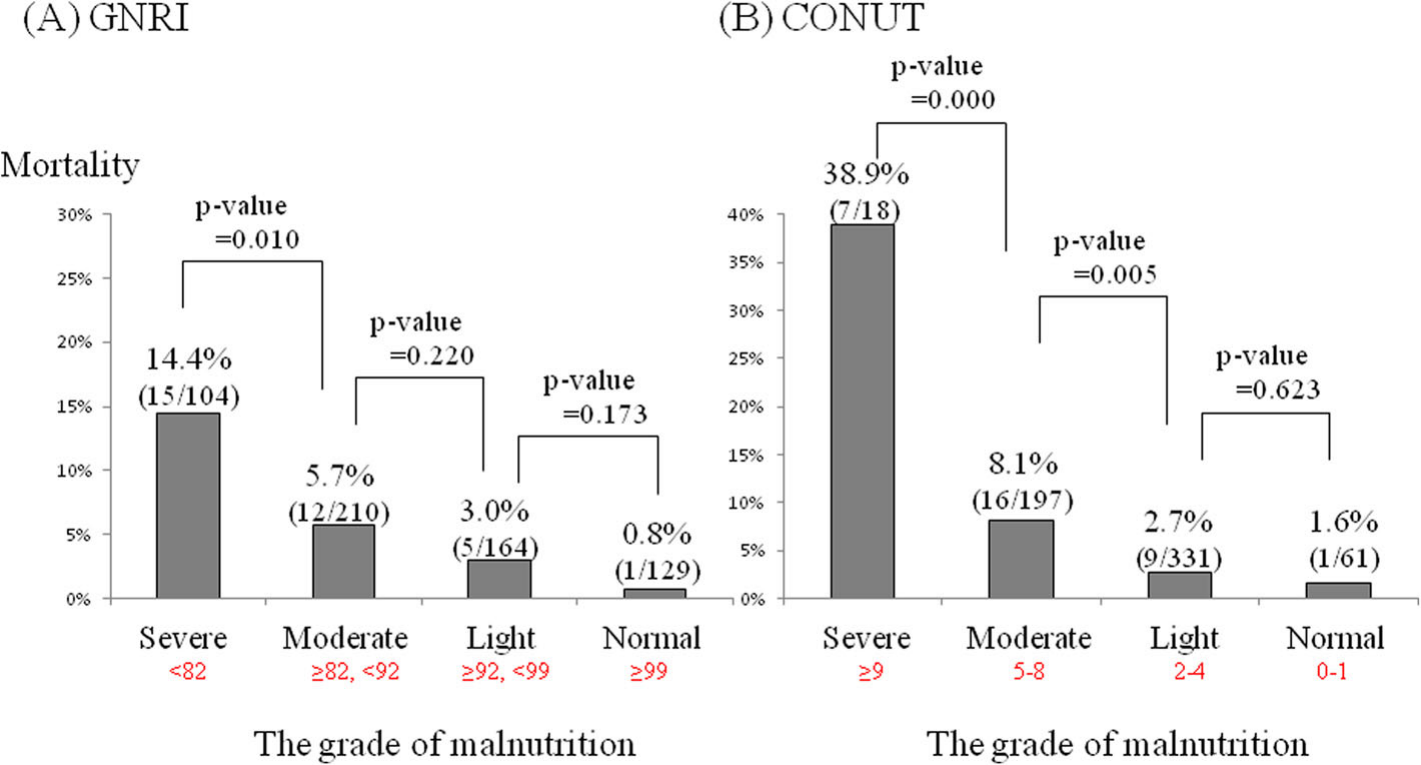
The relationship between the grade of malnutrition and the proportion of 180-day mortality rate in each risk model is shown. In the GNRI, the mortality rates in the severe group (< 82), moderate group (≥ 82, < 92), low group (≥ 92, < 99), and normal group (≥ 99) were 14.4%, 5.7%, 3.0%, and 0.8%, respectively (**a**). In the CONUT, the mortality rates in the severe group (≥ 9), moderate group (5–8), low group (2–4), and normal group (0–1) were 38.9%, 8.1%, 2.7%, and 1.6%, respectively (**b**)

Figure 1 illustrates the relationships between the grade of malnutrition and the 180-day mortality. In the GNRI, the 180-day mortality rate in the severe group, moderate group, low group, and normal group was 14.4%, 5.7%, 3.0%, and 0.8%, respectively (Fig. 1a). The 180-day mortality in the severe group was significantly higher than that in the moderate group (*p* = 0.010). In the CONUT, the 180-day mortality rate in the severe group, moderate group, low group, and normal group was 38.9%, 8.1%, 2.7%, and 1.6%, respectively (Fig. 1b). The 180-day mortality in the severe group was significantly higher than that in the moderate group (*p* = <0.001) and the mortality in the moderate group was significantly higher than that in the low group (*p* = 0.005).

**Fig. 2:**
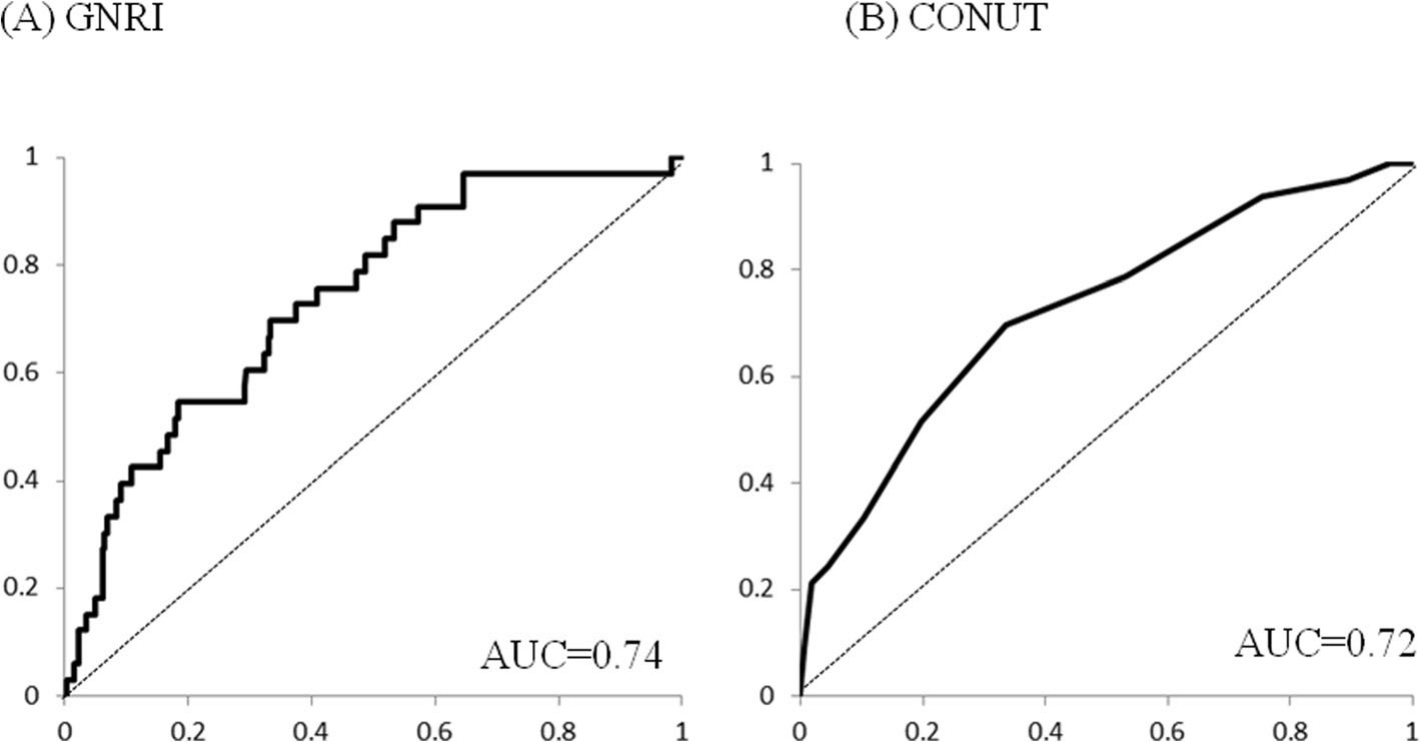
The area under the receiver operating characteristic curve (AUC) to predict the 180-day mortality for each risk model is shown. The AUC values were 0.74 for the GNRI (**a**) and 0.72 for the CONUT (**b**)

Figure 2 shows the AUC to predict the 180-day mortality for each model. The AUC values were 0.74 for the GNRI and 0.72 for the CONUT.

**Fig. 3:**
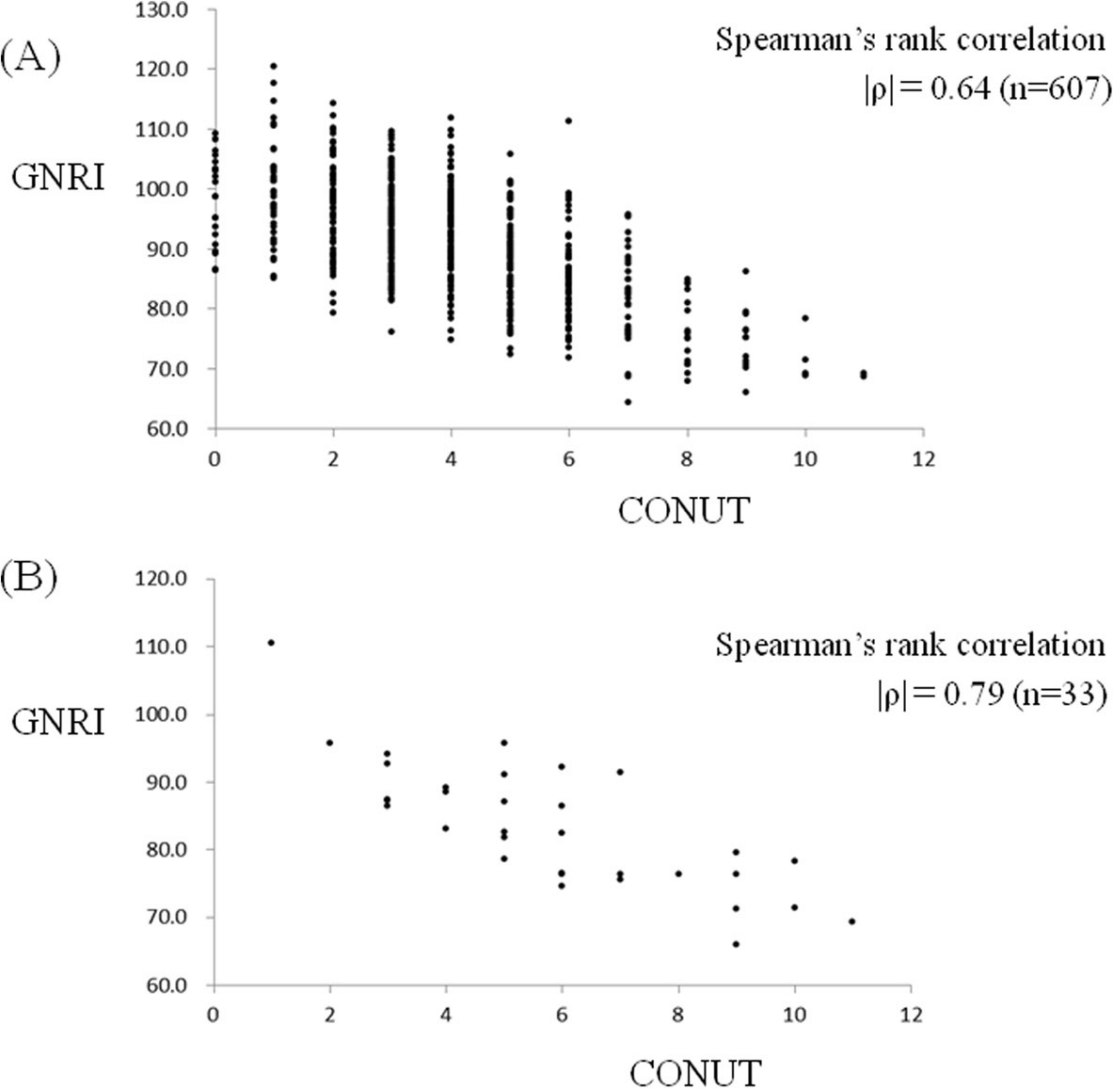
The correlation analyzed Spearman’s rank correlation test between the GNRI and the CONUT is shown. The *ρ* value was 0.64 in the total of 607 patient series (**a**) and that was 0.79 in the 33 non-survivor group (**b**)

Figure 3 shows the correlation between the GNRI and the CONUT. The *ρ* value between these two models was 0.61 in the total of 607 patients and was 0.78 in the 33 non-survivors.

## Discussion

Our present retrospective findings indicate that malnutrition is a significant risk factor for the 180-day mortality following a hip fracture surgery. Generally, malnutrition has been reported to be associated with higher rates of postoperative complications, postoperative mortality, and readmissions in hip surgery [9, 10]. Malnutrition is related to delayed bone healing, surgical site infection, and impaired functional recovery. It is critically important not only as a risk factor for hip fracture, but it also reduces the patient’s ability to recover the functional capacity to the level before fracture [11]. Hypoalbuminemia (albumin level below 3.5 g/dl) is considered to be associated with a higher prevalence of sepsis, and it also had a relative risk of dying of 1.52 (95% confidence interval 1.37–1.70) [10]. Furthermore, it was also noted that serum albumin level less than 3.6 g/dl was associated with a 4-year mortality nearly six times greater (odds ratio 5.85, 95% confidence interval 2.3–16.5) [12]. In a malnourished patient, the immune system is weakened [13]. Therefore, malnutrition is noted to be associated with an increase incidence of death as a result of infection [14]. Also in our present study, 23 of the 33 non-survivors died of infection-related disease. Therefore, malnutrition is closely related to the postoperative mortality and the assessment of nutritional status in patients with hip fractures has become increasingly important. However, the ideal tool to evaluate nutritional status objectively in such patients is controversial. Then, we validated the usefulness of the GNRI and the CONUT as an ideal tool to assess a patient’s nutritional status and to predict the postoperative mortality.

The GNRI based on the two variables was developed to evaluate malnutrition and related morbidity and mortality in hospitalized elderly patients [6]. The GNRI has been reported to be a useful tool to predict surgical site infection after pancreaticoduodenectomy [15], nutritional status and clinical outcomes in peritoneal dialysis patients [16], and clinical outcomes in patients with pyogenic liver abscess [17]. On the other hand, the CONUT based on the three variables was developed to detect malnutrition and assess nutritional status in hospitalized patients [7]. The CONUT has been reported to be a novel biomarker to predict survival rate in patients with esophageal cancer [18], 5-year cancer-specific survival in patients with gastric cancer [19], and recurrence-free survival and cancer-specific survival in patients with renal cell carcinoma [20]. The GNRI and the CONUT have been validated in many clinical fields since 2005; however, to our knowledge, this is the first report to evaluate the usefulness of the GNRI and the CONUT in patients with hip fractures.

Our present findings demonstrate that, in both two models, the 180-day mortality in the severe malnutrition group was statistically high. Additionally, the AUC values to predict the 180-day mortality for these two models were high. In the other previous reports, the AUC values to predict the mortality for the GNRI were 0.77 in the patients with pyogenic liver abscess [17] and 0.75 in the patients with chronic heart failure [21]. In the present study, the AUC value for the GNRI was 0.74, and it was almost the same as those in the previous reports. In the present study, the AUC value for the CONUT was 0.72, which was higher than 0.64 to predict the 5-year survival of patients with colorectal cancer [22]. Therefore, our present retrospective findings showed that the evaluation of a patient’s nutritional status with these two models was useful to predict the postoperative mortality in a patient with a hip fracture.

Serum albumin level is an indicator of protein reserves [18] and included in the equations for calculating both GNRI and CONUT. The ratio of the present to the ideal body weight in the GNRI and the total cholesterol level in the CONUT are considered to reflect caloric depletion [18]. Low cholesterol level is related to be a progress of malignant solid tumor [23]. Therefore, the CONUT is often used to assess a patient’s nutritional status with a malignant tumor. The lymphocyte count in the CONUT is thought to be an indicator of impaired immune defenses secondary to malnutrition [18]. In our present study, patients with acute phase of fractures were eligible; therefore, it would be expected that the ratio of neutrophil count would get larger and the ratio of lymphocyte count would get smaller. Then, we made a hypothesis that the assessment of nutritional status by the CONUT would not be so accurate. However, the AUC value for the CONUT in our patients was higher than that in the patients with malignant tumors. Thus, we consider that the COUNT can be applied for not only malignant disease but also an acute disease like hip fracture.

Our results demonstrated a significant correlation between the GNRI and CONUT. Both of these two models’ values require serum albumin level as a parameter, and serum albumin level plays an important role in their scores. Therefore, we consider that Spearman’s rank correlation between these two models was high statistically. We recommend that clinicians will evaluate a patient’s nutritional status with these two models as a double-checking system. Additionally, we consider that to evaluate a patient’s nutritional status with the GNRI or the CONUT will be a good indicator for introducing preoperative nutritional intervention. Nutritional intervention using oral nutritional supplements can induce an increase in total energy, protein, and lipid intake [24]. Higher nutritional intake is noted to be associated with a lesser prevalence and intensity of delirium, a lower production of oxidative stress-derived products, and a decrease in the incidence of pressure ulcers [25]. Therefore, nutritional intervention is considered to be associated with lower short- and long-term mortality rate [26]. When a patient’s nutritional status is evaluated as moderate or severe grade by using the GNRI or the CONUT, nutritional intervention is recommended as early as possible to improve his or her surgical outcome.

The presence of dementia is also noted to be one of the risk factors related to postoperative mortality after hip surgeries [3, 4]. Also in our present findings, we observed a frequent presence of dementia in the non-survivors group. It is reported that dementia is related to the incidence of surgical site infection, postoperative delirium, and pneumonia; therefore, dementia itself is considered to be one of the risk factors of surgical outcomes [27]. On the other hand, dementia is reported to be an independent risk factor for malnutrition and the odds ratio is noted to be 2.14 (*p* = 0.001) [28]. Therefore, we cannot deny the possibility that dementia was associated with the malnutrition in our non-survivors group. We should keep in mind that there is a strong relationship between dementia and malnutrition in elderly patients.

Our study has several limitations. First, the sample size of patients was relatively small, and the data reflect the clinical outcomes at a single center. Our findings may thus not be generalizable for patients with hip fractures as a whole. Further studies with larger numbers of patients at multiple centers are needed to test our findings. Second, this study was a retrospective investigation, and prospective studies could be informative.

## Conclusions

The results of our analyses suggest that the GNRI and the CONUT are useful to predict postoperative mortality in patients who undergo hip surgery. The calculation of the GNRI and the CONUT is simple, and the variables of these two models are derived from routine preoperative data available at any hospital. We suggest that the GNRI and the CONUT is an easy-to-use model for clinicians.

## Data Availability

The datasets analyzed during this study are available from the corresponding author on reasonable request.

## Abbreviations

AUC: Area under the receiver operating characteristic curve
GNRI: Geriatric Nutritional Risk Index
CONUT: Controlling Nutritional Status Score
